# Implicit Timing as the Missing Link between Neurobiological and Self Disorders in Schizophrenia?

**DOI:** 10.3389/fnhum.2016.00303

**Published:** 2016-06-20

**Authors:** Anne Giersch, Laurence Lalanne, Philippe Isope

**Affiliations:** ^1^Department of Psychiatry, INSERM U1114, Fédération de Médecine Translationnelle de Strasbourg, Strasbourg University HospitalStrasbourg, France; ^2^Institute of Cellular and Integrative Neurosciences (INCI), CNRS UPR 3212, Strasbourg UniversityStrasbourg, France

**Keywords:** time, agency, sequencing, Simon effect, motor control

## Abstract

Disorders of consciousness and the self are at the forefront of schizophrenia symptomatology. Patients are impaired in feeling themselves as the authors of their thoughts and actions. In addition, their flow of consciousness is disrupted, and thought fragmentation has been suggested to be involved in the patients’ difficulties in feeling as being one unique, unchanging self across time. Both impairments are related to self disorders, and both have been investigated at the experimental level. Here we review evidence that both mechanisms of motor control and the temporal structure of signal processing are impaired in schizophrenia patients. Based on this review, we propose that the sequencing of action and perception plays a key role in the patients’ impairments. Furthermore, the millisecond time scale of the disorders, as well as the impaired sequencing, highlights the cooperation between brain networks including the cerebellum, as proposed by Andreasen (1999). We examine this possibility in the light of recent knowledge on the anatomical and physiological properties of the cerebellum, its role in timing, and its involvement in known physiological impairments in patients with schizophrenia, e.g., resting states and brain dynamics. A disruption in communication between networks involving the cerebellum, related to known impairments in dopamine, glutamate and GABA transmission, may help to better explain why patients experience reduced attunement with the external world and possibly with themselves.

## Introduction

Understanding how neurobiological and cognitive disorders in schizophrenia lead to clinical symptoms like self disorders is a crucial step towards understanding the link between genetic and molecular mechanisms on one hand, and the most integrated aspects of our psychic life on the other. Here we focus on timing and on the cerebellum as a point of convergence between several lines of exploration of both sensory and motor aspects related to the self in schizophrenia patients. Our analysis of the literature, especially concerning possible convergence between observations in the motor and timing domains, indeed suggest a common explanation and the involvement of a brain network that includes the cerebellum. First, we summarize data on agency, i.e., the feeling of being the author of one’s own actions, followed by data on timing, before discussing a possible explanation for the results and their clinical implications.

## Agency Disorders in Patients with Schizophrenia

### The Inverse and Forward Models

Delusions of control represent one of the most striking symptoms of self disorder in patients with schizophrenia and were categorized as a first rank symptom by Schneider (1955). When suffering from such delusions, patients can feel they are controlled or influenced by other agents, and these agents can be as varied as god, the television, or extraterrestrial beings. They thus misattribute their thoughts and actions to an external force, and this misattribution is interpreted as an impairment of agency, i.e., an impaired ability to feel one is the agent of one’s own thoughts and actions.

Seminal work by Frith (2005) has led to heuristic hypotheses linking brain dysfunctions and agency disorders observed at a clinical level. Frith’s hypotheses were based on theories resulting from the exploration of motor control, and especially the “internal models” (“inverse” and “forward”). Both “inverse” and “forward” models are necessary to translate intentions into actions. The inverse model is used first, before the execution of the action. The input of the inverse model is the sensory expectations related with the desired goal, and the associated motor commands that have been learned through development (e.g., drinking from a glass is associated with motor commands in the arm). In order to achieve the desired goal, the inverse model predicts the consequences of the learned motor command before its execution and adjusts it on the basis of the current state of the body (Wolpert et al., 1998). The forward model is fed with a copy of the final version of the motor program (the efference copy) and predicts its sensory consequences during the action (von Holst and Mittelstaedt, 1950). Again the motor program is adjusted in case of a mismatch between expected and real sensory consequences.

The comparison between the expected and actual sensory feedback has also been proposed to play a role in the sense of agency. The sense of being the author of one’s own actions would indeed be reinforced if the match between the expected and actual outcome is reasonable (Frith, 2005).

Several authors have shown that loops of motor control are dysfunctional in schizophrenia, with alterations in the self-attribution of the action in patients. A wide-spread hypothesis states that the sense of agency is disturbed in patients due to an alteration at the level of the efference copy (Franck et al., 2001; Jeannerod, 2009; Synofzik et al., 2010; Voss et al., 2010). As a consequence of a disturbed efference copy, patients would not benefit from the match between the predicted and actual outcome of the action, resulting in a weakening of the sense of agency. The delusional belief of being controlled by an external agent would then develop in the case of additional impairments, e.g., abnormal binding between an action and its effect (Haggard et al., 2003; Frith, 2005). Several points remain unclear, however, and our aim here is to synthesize this literature to try and better circumscribe the nature of the impairments, to better understand how self disorders emerge in schizophrenia.

### Cancellation of Predicted Sensory Signals in the Case of Self-Initiated Actions

Both EEG and behavioral studies show alterations in the mechanisms associated with action planning in patients. Several studies have been based on the fact that the processing of the sensory signal occurring as a result of a willed action is canceled (Shergill et al., 2003; Izawa et al., 2008; Brooks and Cullen, 2013). This would help distinguish between signals resulting from one’s own action and those that are not self-initiated. This distinction is important for several reasons. It may play a role in our sense of self, but is also crucial to our adaptation to environment signals. First, canceling predicted signals would help avoid interference with external sensory inputs, and enable better detection of unexpected signals. Furthermore, sensory cancellation is important for the adjustment of action. Note that in the case of an unexpected stimulation, one has to react reflexively, for example, in order to maintain postural balance and stabilize one’s gaze and head in space (Massion, 1994; Cullen et al., 2011). In contrast, in the case of a voluntary action, the action should not be interrupted by a reflexive reaction, because the planning of the action takes the stabilization of the posture into account. In this case, a corollary discharge is used to cancel the processing of the sensory outcomes of the action, which are predicted by means of the efference copy. This would help avoid processing and reacting to the predicted sensory signals inappropriately. Several authors have suggested that patients with schizophrenia do not show normal corollary discharges but that they are unable to inhibit the sensory signals that result from voluntary actions (Blakemore et al., 1998; Shergill et al., 2005). For example, Shergill et al. (2005) designed an elegant paradigm to check for inhibition of haptic feedback. They applied a constant force to the top of the subject’s left index finger, by means of a torque motor and a force transducer attached to a lever. The subjects were instructed to reproduce this force by pressing the lever with their right index finger for 3 s, while the force transducer still rested on their left index finger. The inhibition of the sensory feedback led subjects to underestimate the force they produced as revealed by compensatory overproduction of the force applied on the lever with the right finger (Shergill et al., 2003). Patients with schizophrenia showed the same profile, but the effect was significantly reduced in amplitude (Shergill et al., 2005). Abnormal corollary discharges have also been suggested for eye movements (Thakkar et al., 2015), speech (Ford et al., 2007) and even simple tones (Ford et al., 2014). Interestingly the two latter studies used EEG, thereby confirming inhibition of the auditory signal in healthy volunteers (N1 suppression, meaning a reduction in the amplitude of the evoked potential observed 100 ms after speech onset, Wang et al., 2014). They also showed that EEG activity in the delta/theta range preceding speech predicted N1 suppression. Activity preceding speech can be attributed to action preparation and is believed to correspond to the production of the efference copy. These phenomena, i.e., activity preceding speech onset and subsequent N1 suppression, were all impaired in patients (Ford et al., 2007, 2014). These authors suggest that something is impaired in the patients’ preparation of the action and in their ability to suppress predictable signals. However, whether the impaired cancellation of the predicted sensory inputs result from an alteration of the corollary discharge, a sensory dysfunction or from a temporal mismatch in cortical networks remains to be clarified.

It appears that the efference copy is at least partially preserved. First, it has been shown that patients with schizophrenia are able to automatically adjust their action in the case of an unexpected sensory distortion (Fourneret et al., 2001; Knoblich et al., 2004; Jeannerod, 2009). Moreover, when the patients had to use a manipulandum to resist an imposed collision with a pendulum, they normally adjusted their grip force while looking at the falling pendulum (Delevoye-Turrell et al., 2003). It is noteworthy that in this experiment, the adaptation was explicitly required from subjects. Grip force was also scaled to force impact in the case of voluntary hits on the pendulum. In addition the inhibition of the sensory signals resulting from self-initiated actions is reduced but not suppressed (Shergill et al., 2005; Ford et al., 2007, 2014). These results question the nature of the impairment at the level of the efference copy. Furthermore, a series of impairments has been observed even when the task does not require subjects to consciously attend to their action (Ford et al., 2014; Thakkar et al., 2015). For example, Ford et al. (2014) asked subjects to press a button, and this key press was associated with a tone occurring simultaneously with the key press. The task did not require any subjective judgment from the subjects, but both the evoked potentials observed before the key press (the lateralized readiness potential [LRP]) and those observed after the key press (the N1) were reduced in amplitude in patients relative to controls. These results have been interpreted in terms of a deficit in both the efference copy (thought by the authors to be reflected in the decreased LRP) and the corollary discharge, resulting in a decrease in the subsequent sensory suppression (the N1 suppression), similar to that described regarding speech (Ford et al., 2013). The decrease in LRP may or may not reflect the efference copy, but suggests that action preparation mechanisms are impaired in patients with schizophrenia. Observations with a similar EEG paradigm (Whitford et al., 2011) provided an alternative explanation. This time the sound occurred simultaneously, 50 ms, or 100 ms after the key press. As in previous studies, the authors recorded the suppression of the evoked potential N1, which is assumed to occur as a consequence of the corollary discharges. N1 suppression with 0 delay was decreased in patients relative to controls, as in previous studies. Interestingly however, N1 suppression observed in patients at 50 ms delay had the same amplitude as the N1 suppression observed in controls at 0 ms delay. This may mean that a corollary discharge is sent normally, but arrives too late. This would be consistent with the literature showing abnormal connectivity in schizophrenia. Indeed, a large body of literature suggests that the connectivity between brain areas is impaired in patients, as proposed by Friston and Frith (1995). This is true both during tasks (Uhlhaas and Singer, 2010; Sheffield and Barch, 2016) and during resting states, when subjects relax in the fMRI without doing any specific task (Greicius, 2008; Zhou et al., 2010; Salomon et al., 2011; Hahamy et al., 2014). Connectivity disorders may be associated with structural abnormalities in white matter (Kochunov and Hong, 2014; Wheeler and Voineskos, 2014; Sun et al., 2015), and would be diffuse rather than localized (Zhou et al., 2010). Even if the findings are heterogeneous (Liang et al., 2006; Zhou et al., 2010; Sheffield and Barch, 2016), the outcome would in all cases be less efficient communication between different areas of the brain. Such communication impairment may affect the possibility to send information from one brain location to another, like the corollary discharge.

Once again however, the possibility that the corollary discharge arrives too late due to abnormal neuronal connectivity may not be enough to account for the difficulties the patients face. First, why the above-mentioned potentials preceding the action are abnormal would have to be explained. It is not only the signal transmission that is altered, but also the processing that precedes this transmission. Second, a delay in the inhibition of the sensory processing probably does not explain results like those obtained by Shergill et al. (2005). Recall that, in this experiment, the subjects had to reproduce a force by pressing on a lever. The important point is that they had to press on the lever for 3 s. Hence a 50 ms delay in the corollary discharge probably does not explain the deficit in inhibition observed in Shergill’s study. Other factors may account for the discrepancies in the literature, for example other results that reveal specific abnormalities during action sequences (Zalla et al., 2006; Delevoye-Turrell et al., 2007).

### Abnormalities in Action Sequencing

Studies that explore action sequencing show impairments independent of subjective judgments, and suggest that the planning of a sequence of action is specifically altered in patients with schizophrenia. Delevoye-Turrell et al. (2007) used a load cell to time the subjects’ actions when they were asked to tap their finger on the surface of the cell (“finger tapping” task). To tap on a surface, subjects have to lower and then lift their finger, and the action is thus composed of a sequence of two motor elements. Such a sequence is different from sequences used to consciously execute series of actions, like tapping successively on a blue, red and yellow target. Contrary to such serial reaction tasks, subjects do not necessarily consciously break down a tapping action into two actions. On the contrary, tapping can be considered as one single action, with a single word to describe the sequence. Nonetheless, subjects plan the whole sequence beforehand (Billon et al., 1996). Planning allows them to anticipate the sensory feedback. Indeed, contact with the surface is usually shorter than the time required for the sensory signal to reach the brain and for a motor command to be sent back (Billon et al., 1996; Delevoye-Turrell et al., 2007). This means subjects can lift their finger immediately after contacting the surface. Patients, in contrast, appear not to be able to plan such a simple sequence efficiently (for evidence of planning deficits in patients, see also Jogems-Kosterman et al., 2001). Their contact time with the surface is abnormally long, as if they were waiting for sensory information regarding surface contact before lifting their finger (Delevoye-Turrell et al., 2007, 2012). This is not due to a non-specific slowing down in patients, since they react normally when the load cell is slipping out of their hand. In that case they reflexively grasp the cell as fast as controls (for a more detailed discussion, see Delevoye-Turrell et al., 2007). Finally, abnormally long intervals between elements of the action have also been observed in simple lifting actions. It has been shown that after grasping an object, patients briefly push it down before lifting it (Delevoye-Turrell et al., 2003). All these impairments thus occur in very simple actions that are performed every day and do not require special attention. It has also been suggested that the alterations summarized above are independent of the anti-dopaminergic treatment given to patients with schizophrenia, because they differ from those observed when dopamine is lacking, e.g., in patients with Parkinson’s disease (for a thorough discussion on these points, see Delevoye-Turrell et al., 2003).

All in all, these results were interpreted as a deficit in sequencing motor elements. If this is true, one can ask whether this deficit is an alternative explanation for the impairments reviewed here in patients with schizophrenia. A sequencing impairment may explain why only some aspects of motor preparation and sensory prediction are impaired. Simple movements would be planned correctly and their sensory consequence predicted. It is the smooth sequencing of actions that would be impaired, as well as the prediction of the chain of sensory consequences. This may explain several results in the literature. In paradigms in which a key press is associated with a sound, the key press is already a sequence. Patients need a longer time to lift their finger. They wait for the haptic feedback to launch the second part of the pressing action. This would mean that the action is not planned as a whole but bit by bit. If this is the case, the corollary discharge regarding the sound would be sent only when launching the second part of the pressing action. It would thus be sent later in patients than in controls. The late sending of the corollary discharge may be added to the probable slowing down of the transmission of the message. Moreover, difficulty in planning the action sequence may explain why the preparation of the action is deficient and not only the suppression of the predicted sensory signal (see also Jogems-Kosterman et al., 2001; Delevoye-Turrell et al., 2006). In all, patients may have difficulty sequencing pressing actions and sensory processing. This difficulty may be related to timing difficulties. It is indeed remarkable that difficulties are mainly revealed in abnormally long delays between motor elements (Delevoye-Turrell et al., 2007), and that corollary discharge appears to be delayed rather than suppressed (Shergill et al., 2005; Ford et al., 2007, 2013, 2014). Moreover, sequencing implies ordering of motor elements. These results suggest that time, and especially temporal order, plays a role in these abnormalities. Miall et al. (1993) emphasized the temporal dimension of the forward model, as did Waters and Jablensky (2009) more recently. It is noteworthy that the temporal distortion of the sensory feedback affected patients with schizophrenia both with and without delusions of control (Franck et al., 2001). This suggests that temporal difficulties exist prior to agency disorders. This observation suggests that timing disorders cause at least part of the abnormalities observed in motor planning and execution. The possibility of a timing disorder is reinforced by recent results, which indeed suggest that patients may have serious difficulty ordering events.

## Time Disorders in Patients with Schizophrenia

**Figure 1:**
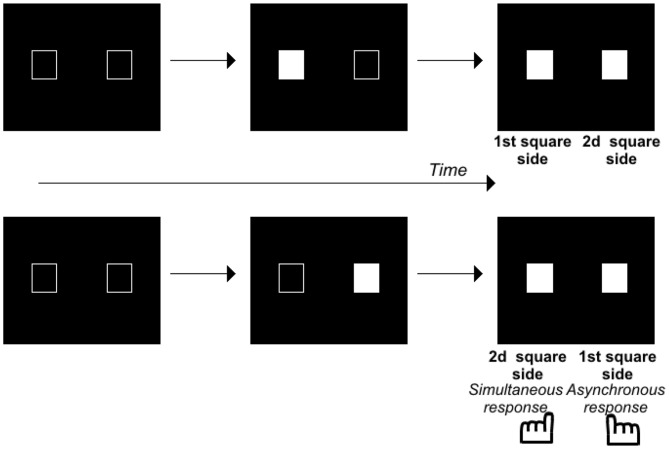
**Illustration of the paradigm used to evaluate the Simon effect.** Two squares were displayed on a computer screen with an asynchrony between 0–100 ms by 8 or 17 ms steps. The subjects decide whether the stimuli are simultaneous or asynchronous and press the left or right response key, respectively. At large asynchronies, the Simon effect shows itself as a tendency to press the key on the same side as the second stimulus, irrespective of the side the stimulus appeared. At small asynchronies, the Simon effect reveals the same tendency in healthy volunteers, but a tendency to press the key on the first stimulus side in patients with schizophrenia.

Here we focus on the literature concerning infra-second time scales, because this scale is particularly pertinent for motor control and the ability to sequence motor elements smoothly. Several results have shown that patients with schizophrenia have difficulty tapping in rhythm with a sound and in detecting rhythm irregularities (Bourdet et al., 2003; Carroll et al., 2009; Turgeon et al., 2012). Duration judgments have also been shown to be impaired, even infra-second durations, i.e., 300–600 ms (Carroll et al., 2008). Furthermore, impairments have been observed for shorter durations that are closer to those required for smooth sequencing, i.e., <100 ms. Durations are no longer perceived for 50 ms intervals (Wittmann, 2011) but successive events can still be distinguished in time. This ability appears to be impaired in patients with schizophrenia. Several studies have indeed shown that these patients have difficulty detecting asynchronies between visual stimuli (Foucher et al., 2007; Giersch et al., 2009; Schmidt et al., 2011; Lalanne et al., 2012a) auditory stimuli (Foucher et al., 2007) and audio-visual stimuli (Foucher et al., 2007; Martin et al., 2014). The difficulty is even greater when patients have to decide on the order of the stimuli, and not only to detect an asynchrony (Capa et al., 2014). Such difficulties may already account for some timing confusions and difficulties in sequencing events (Synofzik et al., 2010; Whitford et al., 2011), although this is not the only difficulty identified. Lalanne et al. (2012a) indeed showed that healthy subjects can distinguish events in time at an automatic level even when they judge them to be simultaneous. They used the Simon effect to detect automatic responses to time delays, independent of subjective judgments. The Simon effect is defined by a tendency to press the key on the same side as the stimulus displayed on the screen, even when the subject of the task does not require processing of the spatial position of the stimuli. This effect was used as a tool to measure the implicit, automatic processing of the stimulus sequence (Figure 1; for a detailed discussion on the mechanisms underlying the Simon effect, see Hommel, 2011a,b; Van der Lubbe and Abrahamse, 2011). When stimuli are simultaneous, each is displayed on one side of the screen at the same time. In that case, information is perfectly symmetrical and the response cannot be biased on either side. When there was a clear asynchrony, however, Lalanne et al. (2012a) showed that all subjects tend to press the key on the side of the second visual stimulus (irrespective of whether it is on the right or on the left side, Lalanne et al., 2012b). Importantly, this bias to the side of the second visual stimulus is also observed in healthy subjects when they do not perceive an asynchrony between stimuli (Lalanne et al., 2012a,b). This bias has been suggested to reflect an ability to predict and follow visual events over time (Poncelet and Giersch, 2015). This occurs over time intervals as short as 17 ms. The same method was thus used to analyze results in patients with schizophrenia. Patients also show a Simon effect (Figure 1), even for time intervals as short as 8 and 17 ms (Lalanne et al., 2012a,b; Giersch et al., 2015). However, instead of being biased to the side of the second square, they were biased to the side of the first square for asynchronies of 8 and 17 ms (again, this effect was observed independently of the side on which the first square appeared, see Giersch et al., 2015).

The results suggest that patients do not lack temporal accuracy, inasmuch as they distinguish stimuli in time, at least implicitly. However, they appear to have difficulty predicting/following events over very short time intervals, as if they are stuck on the first event, and unable to move smoothly from one event to the other. This difficulty has been shown to correlate with the patients’ difficulty in detecting an asynchrony and in ordering visual stimuli (Giersch et al., 2015). This suggests that the abnormalities observed at 8 ms are related to difficulties in processing the sequence of two events. Two asynchronous and successive events indeed represent a sequence of events. This is where these results converge with those described on motor actions. Difficulty in processing a sequence of two visual stimuli indeed resembles the difficulty schizophrenia patients have sequencing motor elements. The ability to automatically sequence perceptual events would be impaired in patients, in addition to their difficulty in sequencing motor elements. It should be noted here that this difficulty also resembles the difficulties observed with “Rapid Serial Visual Presentation”, i.e., when subjects have to process two stimuli successively in a sequence of pictures (Mathis et al., 2011). However, in these paradigms, each stimulus is shown for 50–100 ms, and the time scale of the phenomena is thus different. Further studies are required to check for a possible relationship with the ability to predict/follow events at a shorter time scale.

The fact that both perception and action impairments raise hypotheses concerning a sequencing deficit at short time scales suggests that the two impairments may have the same origin. This is all the more plausible that a common brain structure is responsible for different types of sequencing. The cerebellum is indeed believed to play an important role in sequencing, not only for motor programming, but also for cognitive functions (Leiner et al., 1993; Ito, 2008; Koziol et al., 2014). The possibility that common mechanisms underlie motor control and more high-level cognition or perception is not as surprising as it may seem at first sight. Feinberg (1978), and Feinberg and Guazzelli (1999) proposed that mechanisms analogous to corollary discharges are at play during conscious thought. The idea that predictive mechanisms play a pervasive role in many if not all cognitive functions is the basis of embodiment and predictive coding (Friston, 2008). Predictive coding consists in applying the Bayesian statistical theory to brain functioning: cognition would require the integration and exchange of incoming sensory information and expectations, i.e., “priors” in Bayesian terms. This is not sufficient to implicate the cerebellum since predictive coding would apply to all areas of the brain, and corollary discharges may be produced in many different areas of the brain (Crapse and Sommer, 2008). Here however, we are emphasizing predictions regarding sequences of sensory information or motor elements occurring over some milliseconds, and this orients our hypotheses towards the cerebellum. A role for the cerebellum in schizophrenia is not new in the literature. Andreasen (1999) already stressed the importance of the interactions between the cerebellum and other parts of the brain, and especially the basal ganglia, the thalamus, the prefrontal cortex, and the medial temporal lobe, in coordination and time cognitive functions. Andreasen (1999) hypothesized that the alteration of this network would be responsible for a “cognitive dysmetria”, i.e., difficulty in coordinating thoughts akin to the disorganization of thought described at a clinical level in patients. This coordination failure resembles the sequencing difficulty discussed above. It is also noteworthy that at least timing impairments have been shown to be correlated with disorganization in patients with schizophrenia (Foucher et al., 2007; Giersch et al., 2015), which is consistent with Andreasen’s hypothesis. The role of the cerebellum and its interactions with the remainder of the brain are recalled in the following paragraph, and evidence for disturbances at the cerebellar level in patients with schizophrenia is then reviewed.

## Cerebellar Impairments in Schizophrenia

### The Cerebellum, Action Sequencing, and Timing

**Figure 2:**
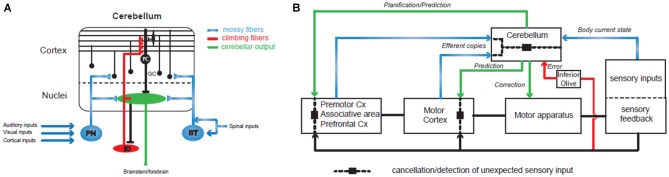
**Involvement of the cerebellum in action sequencing and timing. (A)** Basic organization of the olivo-cerebellar circuit. The cerebellum receives two main excitatory inputs: the mossy fiber pathway (in blue) carrying sensorimotor information from the cerebral cortex via the pontine nuclei (PN), the brainstem (BT) and the spinal cord; the climbing fiber pathway (in red) originating in the inferior olive and conveying integrated signals. Both inputs converge to the Purkinje cells (PC), the sole output of the cerebellar cortex, directly for the climbing fiber or through a relay on the granule cells (GC) for the mossy fibers. **(B)** The cerebellum controls sensorimotor systems at several levels: (1) during the planning of motor programs, prefrontal and premotor cortices send a copy (“efferent copy”) of the plan to the cerebellar hemispheres through the PN and the mossy fibers. Cerebellar inverse internal models of the body adjust the corresponding motor command based on information received through other mossy fiber inputs conveying sensory inputs that build a dynamic picture of the current state of the body (mossy fiber pathways in blue). Ultimately, the prediction is sent back to the forebrain via the thalamus and used to adjust the motor plan. (2) When the motor plan has been validated by the premotor systems, the motor cortex generates the program and sends it both to the motor apparatus for execution and to the cerebellar cortex as an efferent copy that will be at the base of the ongoing control of movement execution. Another prediction through cerebellar forward models and based on the expected sensory feedback is then used to adjust the motor plan online in the motor cortex and to adapt the movement via brainstem nuclei. Therefore, prediction pathways are compared (dashed line and black square) at different levels of motor control with the actual sensory feedback in order to cancel the expected sensory feedback which allows the detection of unexpected events. One type of comparison leads to the emission of an error/unexpected signal through the inferior olive and the climbing fiber pathway (in red) and causes plastic changes in the cerebellar cortex in order to adjust internal models. Since the perception of a movement as being voluntarily executed is linked to an appropriate cancellation of the predicted sensory feedback, dysfunctions in these different comparisons may lead to agency disorders. Internal models of the cerebellar cortex are constantly adjusted through synaptic plasticity controlled by the climbing fiber pathway (in red) that convey information about the sensory outcomes of the motor program.

The cerebellum plays a major role in the control and learning of skilled movements and is at the heart of motor coordination (Thach et al., 1992; Bastian, 2006). The cerebellum can coordinate the action of many motor units in order to perform a wide array of complex movements. Patients with cerebellar dysfunctions (infarct, lesions or degeneration) have specific motor impairments in multi-joint coordination. If the movement is still possible, patients tend to move one joint at a time and break down their movements, leading to inaccuracies such as target overshoot and ataxia or dysmetria (Thach et al., 1992; Bastian, 1997, 2006). It has been shown in primates that inactivating cerebellar nuclei, the output stage of the cerebellum, can reproduce these symptoms during a simple reaching task (Vilis and Hore, 1977). By recording EMG activities, this group demonstrated that agonist and antagonist muscle contraction systematically overshoot during the movement. These findings suggest that the cerebellum controls multi-joints sequencing by predicting interaction torques between joints, and compensates for them during the movement. This hypothesis was confirmed in primates by electrophysiological recordings performed in the cerebellar nuclei, the output module of the cerebellum. During voluntary arm movements, neurons located in the dentate nucleus, which form a close loop with the cerebral cortex (Kelly and Strick, 2003), can discharge before the onset of the movement (Trouche and Beaubaton, 1980; Schieber and Thach, 1985; Thach et al., 1992; Figure 2). In contrast, those located in the interposed nucleus, which communicates with the motor cortex (Hoover and Strick, 1999) discharge mostly during movement execution (Thach, 1978; Harvey et al., 1979). Therefore, the cerebellar hemispheres may be involved in the preparation of the motor program or movement rehearsal and the intermediate part and the vermis may control the ongoing sequencing of movement, posture and gait. Indeed, experiments combining EMG and MEG recordings in humans showed that such multi-joint movements combine activity in the contralateral primary cortex, the premotor cortex, the thalamus and the ipsilateral cerebellum (Gross et al., 2002; Schnitzler and Gross, 2005) and involve a synchronized oscillatory network at 6–9 Hz.

Controlling the sequencing of individual components for a voluntary movement means that the cerebellum has to implement two important tasks: (1) receive an internal copy of the motor program (the efferent copy or corollary discharge; Figure 2); (2) use an internal model of the motor apparatus (forward model) or controller (inverse model; Figure 2; for a review see Wolpert et al., 1998) in order to predict the sensory consequences of unitary events. A wealth of studies have demonstrated that the cerebellum receives an internal copy (the efferent copy or corollary discharge) of the motor command through the mossy fibers, one of the two main excitatory input pathways to the cerebellum (Figure 2A), and can learn to predict the future state of the body (Wolpert et al., 1998; Kawato, 1999; Ito, 2005, 2008; Bastian, 2006; Ebner and Pasalar, 2008; Bhanpuri et al., 2013; Figure 2). Studies in rodents and in primates describe how the Purkinje cell (PC) discharges, the sole output of the cerebellar cortex, correlates with different kinematic features of the movements as required for a forward internal model of the motor apparatus (Angelaki et al., 2004; Popa et al., 2012; Azim and Alstermark, 2015; Chen et al., 2016; Tomatsu et al., 2016). If proper action sequencing relies on forward models stored in the cerebellum, then learning mechanisms must constantly adjust these models as the relations between individual elements of the body (e.g., muscles or joints) change continuously. Indeed, the olivo-cerebellar pathway via the climbing fibers controls plasticity events in the cerebellar cortex underlying adjustments in forward models (Figure 2; Ito, 2005, 2008; D’Angelo, 2014). Such learning properties have been demonstrated in many different paradigms both in rodents and in humans: the gain of the vestibulo-ocular reflex (Jörntell and Hansel, 2006) or the ocular saccades (Dash and Thier, 2014) or simply when throwing a ball at a visual target (Martin et al., 1996). Notably, in the latter experiment patients with a lesion in the olivo-cerebellar system showed impaired prism adaptation during throwing. All these studies assessed the role of the cerebellum in action sequencing, but how and where predictions made by the cerebellum are combined with the motor program is still an open question.

An appealing hypothesis is that the prediction can cancel the self-induced sensory feedback in order to detect unexpected events (see above and Blakemore et al., 1998). Unexpected events which could lead to motor errors would then be corrected and participate in the adjustments of the cerebellar internal models through learning (Diedrichsen et al., 2005; Popa et al., 2012). Indeed, recent experiments in primates (Brooks and Cullen, 2013; Cullen, 2014) demonstrated that the output signal of one cerebellar nucleus involved in the vestibular control of head vs. body motion is canceled during a voluntary movement while neuronal discharge is correlated with movement when passively executed. These elegant experiments demonstrate that the cerebellum receives an efferent copy of the motor program and computes an expected sensory feedback through a forward model of the motor apparatus to cancel the actual sensory feedback. This processing may lead to an emerging property: the differentiation between self- and externally induced sensory signals, a key feature frequently altered in schizophrenia (see above). If the cancellation of an expected sensory feedback underlies the proper sequencing of actions, then the cerebellar control would rely on its capacity to properly time neuronal processes, as suggested by experiments in which patients were asked to draw circles repetitively (Spencer et al., 2003). Cerebellar patients were severely impaired when asked to pause between each circle while continuous drawing was normal. Also, implicit timing is likely to be a key mechanism for appropriate anticipation of the next step in a complex movement or in the cancellation of the re-afferent sensory input (Manto et al., 2012). Patients with ataxia, a family of typical cerebellar diseases, are impaired in the precise timing of individual components involved in rapid movements (Bastian, 1997). Moreover, a classical paradigm of cerebellar learning, eyelid conditioning, is based on event timing and the processing of time. The protocol consists in controlling eyelid closure following a single air puff into the eye. Eyelid closure is a reflex loop, but it can be conditioned by a single tone if the tone and the air puff are repeatedly presented in overlapping intervals (delay conditioning). After learning, eyelid closure is adjusted in time, showing that the air puff is anticipated with very precise timing. This paradigm has been intensively studied in the last four decades both in animal models (McCormick and Thompson, 1984; Yeo et al., 1986; Raymond et al., 1996; Garcia et al., 1999; Longley and Yeo, 2014) and in humans (Logan and Grafton, 1995; Parker et al., 2014). MRI and transcranial magnetic theta burst stimulation of the cerebellar cortex recently confirmed the specific role of the cerebellum in the absolute timing of events (Grube et al., 2010). Patients with cerebellar deficit (lesion or cortical degeneration) are unable to acquire the appropriate timing of the conditioned reflex. Also, an increasing number of studies has demonstrated that deficit in eyelid conditioning is correlated with schizophrenia (Bolbecker et al., 2009; Lubow, 2009; Forsyth et al., 2012). All these data thus confirm the role of the cerebellum in timing and sequencing events. Since these abilities appear to be at least partially impaired in patients, it should follow that the cerebellum plays a role in the patients’ impairments. In the next section we summarize the imaging evidence for the involvement of cerebellum in patients’ difficulties.

### Cerebellar Dysfunction in Schizophrenia?

Several imaging results point to the implication of the cerebellum in the disorders of patients with schizophrenia. First many studies have shown that they suffer from minor neurological disorders and especially discoordination and fine motor movement disorders (Docx et al., 2012; Bervoets et al., 2014; see reviews in Picard et al., 2008; Bachmann et al., 2014). Second, several fMRI studies suggest the involvement of the cerebellum. This is especially the case in resting-state studies. In such states, subjects are assumed to mainly have self-referential thoughts. Inasmuch as reference to self is expected to be disturbed in patients, this makes these studies all the more pertinent to analyze the spontaneous train of thoughts in patients with schizophrenia (Northoff, 2014, 2015). Two studies reported anatomical abnormalities in patients with schizophrenia relative to controls, i.e., a subtle disruption in the middle cerebellar peduncles (Okugawa et al., 2004), or a reduced cerebellar volume (in patients with schizophrenia but not with bipolar disorder, Laidi et al., 2015). However, most observations concerned the connectivity between the cerebellum and other parts of the brain. Transcranial magnetic stimulation of the cerebellum has demonstrated that it influences the dorsal attention system, as well as the default mode network (DMN) whose activation is observed during resting states (Halko et al., 2010). It is thus not surprising that the connectivity between the cerebellum and the DMN has been identified as being disturbed in patients with schizophrenia, with an excess of connectivity in patients relative to controls (Guo et al., 2015; Shinn et al., 2015). Interestingly, Liang et al. (2006) recorded connectivity between all brain regions, and observed that the cases of increased connectivity in patients with schizophrenia relative to controls mainly concerned the relationships between the cerebellum and other parts of the brain. It might seem surprising that connectivity is increased rather than reduced, but increased connectivity is not necessarily optimal. For example, hyperconnectivity involving different pathways has been shown to be associated with hallucinations (Hoffman and Hampson, 2012). Besides, reduced connectivity between the thalamus and the cerebellum has also been described (Wang et al., 2015), which fits well with the hypothesis of Nancy Andreasen (1999) regarding a dysfunctional cortico-cerebellar-thalamo-cortical circuit. It should be noted that some authors have compared connectivity abnormalities in patients with schizophrenia vs. bipolar disorders vs. controls, in various networks including the cerebellum (midbrain-cerebellum, Khadka et al., 2013; cerebellum-precuneus, Rashid et al., 2014). These studies revealed abnormalities in patients with schizophrenia that did not generalize to patients with bipolar disorders, which suggests these findings might have some specificity. Moreover, recent techniques aimed at exploring the dynamics of cerebral connectivity and activation during resting states, by computing connectivity on successive time windows (Hutchison et al., 2013). This means that instead of recording connectivity throughout the scan session, what is observed is how connectivity patterns evolve dynamically with time. Ma et al. (2014) used this technique to explore connectivity in patients with schizophrenia vs. controls. They showed that the patterns of spatial concordance fluctuate more in patients with schizophrenia than in controls, and especially the patterns between fronto-parietal, temporal lobe, and cerebellar regions. At the present time, it is difficult to derive the exact functional implications of cerebellar disorders from these studies. We have seen above that patients with schizophrenia show high temporal accuracy at an automatic level, suggesting that not all functions of the cerebellum are impaired. The fact that impairments mainly concern connectivity between the cerebellum and other parts of the brain certainly fits the hypothesis that the cerebellum is not severely impaired *per se*. Unlike patients with cerebellar lesions, patients with schizophrenia do not show a marked slowness in gripping objects (Delevoye-Turrell et al., 2003) and symptoms that remind of cerebellum lesions are rather discrete, e.g., a slight effect on postural sway (Kent et al., 2012). Even though the regions of the cerebellum that are responsible for sequencing and timing differ from those involved in gait and posture, it is highly unlikely that cerebellum lesions alone account for impairments in schizophrenia. As suggested by Andreasen (1999), the involvement of a network including the cerebellum is more likely. We propose that the sequencing role of the cerebellum and how these sequences are integrated at a conscious level may represent a critical process in the organization of actions, perceptions, and possibly thoughts in general. This could affect several aspects of the self, as reviewed in the following.

## Clinical Implications

The idea of a link between perception, action, and self-disorders is not new. Unusual perceptual experiences have been described during prodromal phases of schizophrenia, and may be at the origin of the feelings of depersonalization observed during the emergence of schizophrenia (Parnas and Handest, 2003; Uhlhaas and Mishara, 2007; Mishara and Fusar-Poli, 2013; Postmes et al., 2014). Fletcher and Frith (2009) proposed that unusual perceptual experiences might be subtended by a disturbance in predicting and updating beliefs about the world. The originality of the present work is to approach this question through the time perspective. The re-reading of the literature together with our own experimental data suggests that connectivity disorders in a network including the cerebellum, but also for example, the thalamus and the frontal cortex, lead to perception and action fragmentation, and ultimately to self disorders. Elsewhere, we reviewed how a difficulty in processing trains of sensory information in a continuous way may have impact on how we feel being ourselves relative to the outer world (Martin et al., 2014). Here we only summarize the main ideas along these lines. One basic idea is that the sense of self would require that sensory information is felt as being continuous in time. It has been proposed since long that the sense of temporal continuity relies on the ability both to retain information in mind and to predict what will happen next. One famous example is the example of the melody (Husserl, 1991). When we hear music, at a given instant, we have both the preceding, the present and the future note in mind. This implies we are capable of integrating past, present and future, and hence of linking events in one continuous perception instead of perceiving fragmented, discrete events. At a neuroscientific level, predictive coding (Friston, 2008) may be involved in integrating events over time. Predicting information on a millisecond scale may indeed play the role of automatically linking discrete events into a continuous flow of information. This would make it possible to predict how the environment will be perceived at the next instant. Confirmation of the expected information would then confirm that the outer world is predictable and stable. Conversely, a difficulty in predicting trains of information would disrupt this sense of continuity. This might also apply to our body and eye movements (Wurtz et al., 2011). Feeling oneself as one unique and stable self may indeed require that both interoceptive and haptic perception are stable and reliable. Experimental manipulations aimed at inducing a sensorimotor conflict have been shown to affect the bodily self, i.e., the feeling of our body ownership (Blanke et al., 2015). For example, when subjects touch a virtual wall in front of them while being synchronously touched on the back, the distance between them and the wall is recalibrated, as if their back had moved to the front (Blanke et al., 2014). These results show the importance of haptic information for the sense of self. More generally, action is dynamic and requires the prediction of a train of sensory feedback. The prediction then allows a mismatch to be detected between prediction and actual sensory feedback, and helps recalibrate the movement. This adaptation to the environment would contribute to preserve a sense of stability, inasmuch as our action would be executed smoothly without conscious disruption. Conversely, a dysfunction of these predictive mechanisms might result in the outer world being perceived as unstable. One may wonder whether this briefly happens in patients on some occasions, as suggested by self-reports like the following (cited in Chapman, 1966): “It’s as if you were seeing one picture 1 min and another picture the next. I just stop and watch my feet. If I move, everything alters every minute and I have no control over my legs.” As developed above, we have suggested that disturbances occur in the preparation of motor sequences or perceptual sequences. In real life, preparing an action or a thought requires activating routines of movements or thoughts that were learned at the youngest age. The results summarized in the present manuscript lead us to propose that the integration of such routines in a sequence of actions or thoughts is difficult for patients, even at an elementary level (e.g., when tapping on a surface, or when following visual events over very short periods). This hypothesis may help better understand the precise mechanisms subtending the patients’ difficulties when they have to prepare actions and thoughts, monitor them, and plan coherent behavior in general. The fact that the impairments concern elementary mechanisms that are beyond consciousness may explain, at least in part, that patients cannot report them clearly. They may nonetheless be manifest at a clinical level, and consistent with proposals in the literature (Mishara and Fusar-Poli, 2013), they may ultimately lead to delusions. A disruption in the feeling of continuity may play an important role by affecting the conscious experiences of the patients. Unusual conscious experiences would then lead patients to try to explain these changes. As a result, patients may end up attributing their own actions or thoughts to an external source. These beliefs are diagnosed as delusions of control, one of the main positive psychosis symptoms. In sum, a difficulty in timing sequences of actions and perceptions might be sufficiently destabilizing to lead to different types of symptoms and self disorders.

Our aim here was to provide heuristic hypotheses to link elementary disorders to the most complex and integrated identity disorders encountered in patients with schizophrenia. We developed possible links between dysconnectivity in brain networks including the cerebellum, time disorders and the self. Can we go further by linking these impairments with alterations at the molecular level? Although we have no data pointing to precise molecular and genetic targets, there are some straightforward links between impaired connectivity and dopamine, glutamate and GABA (reviewed in Pittman-Polletta et al., 2015). In addition, abnormal connectivity has been observed in subjects at high risk for schizophrenia (Whalley et al., 2005), which suggests a link with genetic risks. Many candidate genes have been proposed to be involved in abnormal connectivity in patients with schizophrenia (Bellon, 2007; Boland et al., 2007; Olié et al., 2009; Balu and Coyle, 2011; Carlson et al., 2011; Jiang et al., 2013). As all these systems of neurotransmission are connected, genetic variations in one system might simultaneously impact and disorganize gabaergic, glutamatergic and dopaminergic neurotransmission. It remains to be investigated to what extent this abnormal connectivity concerns the cerebellum, and to what extent it accounts for the behavioral and clinical abnormalities described above.

## Conclusion

Many studies have shown motor and perception impairments in patients with schizophrenia that may lead to disorders of the self. These disorders may alter the ability to sequence mental activities, whether action or perception, and may prevent patients from acting in a coherent way, adapting to their environment, and feeling the outer world and themselves as being stable in time. The fact that impairments have been observed at the millisecond level, especially when sequences are involved, suggests the involvement of the cerebellum. The role of sequencing is compatible with the results of many studies, and can lead to impairments that are similar to those that would result from an impairment at the efference copy level. However, the identification of a role for sequences allows us to account for a series of perceptual as well as motor disturbances. This may more easily account for the feeling of strangeness experienced by patients, and their difficulty in following and predicting information smoothly. Furthermore, linking the impairments with a disordered network including the cerebellum allows us to propose how connectivity disorders and related molecular impairments lead to the complex self-disorders observed in patients with schizophrenia. Hopefully future developments regarding the way action is planned will shed further light on patients’ impairments, e.g., by allowing us to better understand how the cerebellum interacts with other areas of the brain and the respective roles played by the vermis and hemispheric regions of the cerebellum.

## Author Contributions

AG, LL, and PI all contributed to the elaboration of the ideas developed in the manuscript. AG wrote the first draft of the manuscript. PI and LL completed parts of the manuscript, regarding the physiology of the cerebellum and the clinical implications respectively. AG, LL, and PI all made critical amendements to the first version of the manuscript.

## Conflict of Interest Statement

The authors declare that the research was conducted in the absence of any commercial or financial relationships that could be construed as a potential conflict of interest.
